# Association between sugar-sweetened beverages and duration of physical exercise with psychological symptoms among Tibetan university students at high altitude

**DOI:** 10.3389/fpsyg.2024.1380893

**Published:** 2024-04-25

**Authors:** Wei Song, Fan Su, Shengpeng Li, Yongjing Song, Guangxin Chai

**Affiliations:** ^1^College of Education and Sports Sciences, Yangtze University, Jingzhou, China; ^2^College of Physical Education, China Three Gorges University, Yichang, China; ^3^School of Preschool Education, Jingzhou Institute of Technology, Jingzhou, China; ^4^School of Physical Education and Health, Jiangxi Science and Technology Normal University, Nanchang, China

**Keywords:** adolescents, psychological symptoms, eating behavior, high altitude, exercise habits

## Abstract

**Background:**

Sugar-sweetened beverages (SSBs) and duration of physical exercise are strongly associated with physical health. Unfortunately, there are few studies focused on the association with psychological symptoms, let alone Tibetan university students at high altitudes in China.

**Methods:**

A stratified cluster sampling method was used to include 8,268 Tibetan university students aged 19–22 years in Qinghai and Tibet, both of which are high-altitude regions of China. Self-assessment questionnaires on SSBs, duration of physical exercise, and psychological symptoms were administered. The chi-square test and logistic regression analysis were used to analyze the associations among them.

**Results:**

The detection rate of psychological symptoms among Tibetan university students in high-altitude areas of China was 16.7%, with in girls (18.2%) higher than that in boys (14.8%), and the difference was statistically significant (*χ*^2^ = 11.73, *p* < 0.01). The proportion of SSBs for university students ≤1 time/week, 2–5 times/week, and ≥ 6 times/week were 54.2, 24.3, and 21.5%, respectively. The proportion of duration of physical exercise for >60 min/d was only 5.4%. Logistic regression analysis showed that compared with the SSBs ≤1 time/week group of university students, SSBs 2–5 times/week (OR = 1.45, 95% CI: 1.24–1.70) and ≥ 6 times/week (OR = 3.06, 95% CI: 2.62–3.57) had an increased risk of psychological symptoms (*p* < 0.001). In the reference group, the risk of psychological symptoms was also significantly increased in the group of university students with duration of physical exercise >60 min/d (OR = 2.08, 95% CI: 1.48–2.93), and the risk of psychological symptoms was also significantly increased in the group with duration of physical exercise <30 min/d (OR = 2.08, 95% CI: 1.48–2.93). The risk of psychological symptoms was also significantly increased in the university students with the duration of physical exercise <30 min/d (OR = 2.08, 95% CI: 1.48 ~ 2.93) group.

**Conclusion:**

SSBs and exercise time may be important influences on the psychological symptoms of Tibetan university students at high altitudes in China. This study has important implications for mental health planning in universities in highland areas and may also provide guidance for mental health interventions for Tibetan university students.

## 1 Introduction

The psychological problems of university students are becoming more and more serious and have a negative impact in many ways, increasing the medical burden on society (Wathelet et al., 2020). The incidence of psychological problems among university students is increasing dramatically and has become an important public health issue of common concern in countries around the world (Sheldon et al., 2021; Herbert, 2022). A survey using the CES-D scale showed that the average depressive symptoms score was 16.24 in university students in the United States, which showed severe depressive symptoms, and more than 50% of university students experienced the 8 most common stressors (Acharya et al., 2018). It was also reported that psychological distress reported by university students was strongly associated with greater suicide attempts (OR = 1.9–2.4, 95% CI: 1.1–3.4) (Liu et al., 2019). A survey of Italian university students showed that the prevalence of anxiety was 35.33% and depression was 72.93%, and the author recommended reducing adverse psychological problems through physical exercise (Villani et al., 2021). However, it is a fact that the prevalence of psychological symptoms among university students continues to increase, spreading from developed to developing countries. A survey of Malaysian university students showed that 27.5% suffered from moderate depression and 34% from moderate anxiety, and there were associations with age, gender, and family income (Shamsuddin et al., 2013). The detection rate of psychological symptoms in Chinese university students was higher in boys (10.7%) than in girls (7.6%) and was strongly associated with time spent exercising muscles (OR: 4.19, 95% Cl: 1.82, 9.61) (Ouyang et al., 2022). Another meta-analysis of Chinese university students also showed that depressive symptoms were moderately associated with suicidal ideation and that special attention should be paid to psychological symptoms among non-medical university students in western China, and more psychological and clinical assistance should be provided (Wang et al., 2017). The occurrence of psychological symptoms in university students will affect the future quality of life, work, and academic level, and bring many negative effects on the health of adulthood. Psychological symptoms of university students who do not receive timely attention and guidance may lead to depression, anxiety, non-suicidal self-injurious behaviors, and even suicidal behaviors. Many studies have confirmed that the factors affecting the occurrence of psychological symptoms in university students are multifaceted, including lifestyle, dietary behavior, exercise behavior, physical activity, sleep quality, and other factors (Nyer et al., 2013; Lu et al., 2022a).

SSBs have proven health hazards and are a significant risk factor for hypertension, diabetes, and all-cause mortality (Qin et al., 2020). However, among the many factors that influence university students’ psychological symptoms, the effect of SSBs on university students’ psychological symptoms seems to have received less attention. Past research has confirmed that excessive consumption of SSBs leads to an increased risk of depression and anxiety compared to adolescents with lower levels of SSBs. In a study of adults in 11 U.S. states and the District of Columbia, the prevalence of poor mental health was found to be 26% higher when SSBs were taken 1 or more times per day compared with no SSBs (95% Cl, 1.11–1.43) (Freije et al., 2021). Another study on adolescents in the United States confirmed that excessive consumption of sugary drinks is an important factor leading to psychological symptoms and suggested that the government should increase the tax on sugary drinks in the future to reduce the intake of sugary drinks among adolescents and safeguard their physical and mental health development (Imamura et al., 2015). Fewer studies have been conducted in Chinese adolescent populations. A survey of Chinese university students confirmed that university students who consumed SSB ≥2 times/week (OR = 2.96, 95% Cl: 2.36, 3.70) had a higher risk of developing psychological symptoms compared with those who consumed SSB <2 times/week (Wang et al., 2022). In addition to some associations between sugary drinks and psychological symptoms, there are relatively more studies on physical exercise time and psychological symptoms. Studies have confirmed that physical exercise can increase IGF-1, PI3K, BDNF, and ERK, and decrease GSK3*β* levels and that it can also enhance the activity of the PGC-1α/FNDC5/Irisin pathway, which leads to neuronal survival and the maintenance of good mental health (De Sousa et al., 2021). Results from a survey of 50,054 Norwegian students aged 18–35 years showed that physical exercise was negatively associated with mental health problems and suicide rates in a dose–response manner, with the strongest effect observed for frequency of physical exercise, with girls with lower levels of physical activity nearly tripling their odds of self-reported depression compared to girls who exercised almost every day as a reference, and stronger effect sizes observed among boys (OR ranging from 3.5 to 4.8) (Grasdalsmoen et al., 2020). It is worth noting that there were large differences in the associations between SSBs and physical exercise time with psychological symptoms, the reasons for which may be related to some differences in the time of the survey, geographic area, and survey group.

However, previous research on SSBs and duration of physical exercise and psychological symptoms in students has mainly focused on developed countries, with fewer studies on university students in developing countries. However, previous studies on students’ SSBs and duration of physical exercise and psychological symptoms have mainly focused on developed countries, with fewer studies on university students in developing countries. Unfortunately, no similar studies have been conducted on Tibetan university students at high altitudes in China. As Tibetan university students have been living in the high altitude area of Qinghai-Tibet Plateau for a long time, due to the special natural environment of the plateau, there is a lack of oxygen and low vegetation coverage all year round; at the same time, there is also a big difference in dietary behaviors, lifestyles, and exercise habits with university students from the plains, so it is necessary to investigate and analyze the psychological symptoms of Tibetan university students in high altitude areas to understand the factors affecting the psychological symptoms, to better promote the development of mental health (Chen et al., 2021; Wu et al., 2023). Analyzing the association between SSBs and duration of physical exercise and psychological symptoms among Tibetan university students at high altitudes in China will provide reference and guidance for psychological promotion and intervention.

## 2 Methodology

### 2.1 Participants

**Figure 1:**
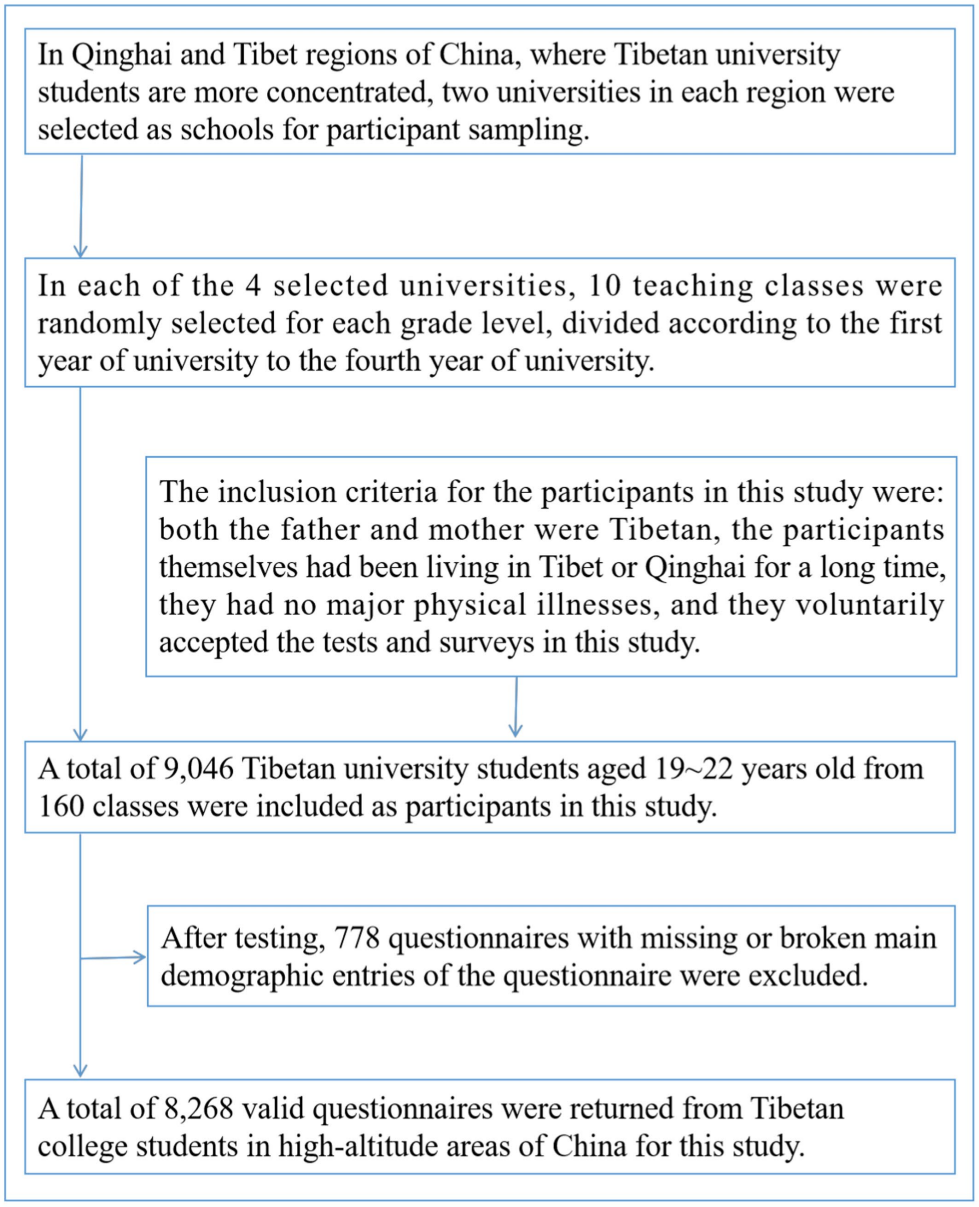
Sampling flow of Tibetan university student participants in high-altitude areas of China.

In this study, participants were sampled using stratified whole cluster sampling. First, in Qinghai and Tibet, China, where Tibetan university students are more concentrated, two universities in each region were selected as the schools from which participants were sampled. Second, in the four selected universities, 10 teaching classes were randomly selected for each grade from the first to the fourth year of university, and all Tibetan university students in the classes served as the participants of this study. The inclusion criteria for participants in this study were: both the father and mother were Tibetan, the participants themselves had been living in Tibet or Qinghai for a long time, they had no major physical illnesses, and they voluntarily accepted the tests and surveys of this study. A total of 9,046 Tibetan university students aged 19–22 years old from 160 teaching classes were included in this study. 778 questionnaires with missing or broken major demographic factors were excluded from the test, and a total of 8,268 valid questionnaires were returned. The mean age of the participants was 20.16 ± 1.03 years. The specific participant sampling process is shown in Figure 1. This study was approved by the Ethics Committee of Jiangxi Science and Technology Normal University (IRB-JXSTNU-2022003).

### 2.2 Basic information

The basic demographic information of the participants was investigated in this study. The main information included the participants’ year of birth, district, school, class, gender, and survey date. The age of the participants was calculated based on the date of birth and the date of the survey. Age was calculated in years, i.e., 9 years means 9.0–9.9 years.

### 2.3 Sugar-sweetened beverages

The survey was conducted using the latest version of a brief questionnaire to assess habitual beverage intake (BEVQ-15) (Hedrick et al., 2012). The main purpose of the questionnaire was to assess participants’ consumption of SSBs in the past 30 days (Fausnacht et al., 2020). The questionnaire mainly assessed participants’ consumption of 15 types of SSBs, including milliliters and frequency of consumption, during the past 30 days. The types of sugary drinks included sports drinks, fruit juice drinks, carbonated drinks, sweetened coffee, sweetened milk, sweetened nut drinks, and sweetened tea drinks. The BEVQ-15 scale on the frequency of consumption of sugary drinks was categorized into >3 times/day, 2 times/day, 1 time/day, 4–6 times/week, 2–3 times/week, 1 time/week, and < 1 time/week, totaling 7 options. Participants made single choices according to their reality. After referring to the literature of previous studies, SSBs in this study were divided into three types: ≤1 time/week, 2–5 times/week, and ≥ 6 times/week, to better analyze the association with psychological symptoms. In this study, each SSBs was calculated in milliliters (mL), and the amount of each calculation was based on the unit of a regular one-fill sugary drink, i.e., 320 mL.

### 2.4 Duration of physical exercise

In this study, the National Survey on Students’ Constitution and Health (CNSSCH) was used to investigate the duration of physical exercise (CNSSCH Association, 2022). The investigation of the duration of physical exercise in this study mainly refers to the duration of the day performing moderate to high-intensity physical exercise, such as ball games, hiking, brisk walking, running, biking, and other sports. The effect of sweating can be achieved during the exercise. This study focuses on the length and frequency of the participant’s participation in various types of physical activity in the past 7 days, including the 5 working days from Monday to Friday and the 2-day weekend on Saturday and Sunday. From this, the average daily hours of participation in physical activity in the past 7 days were calculated. By referring to the classification criteria of international related studies (Zhang et al., 2020), this study categorized the participants into 3 groups based on their average duration of physical exercise in the past 7 days, namely <30 min/d, 30–60 min/d, and > 60 min/d.

### 2.5 Psychological symptoms

In this study, self-reported psychological symptoms were screened using the multidimensional sub-health questionnaire of adolescents (MSQA), which was developed by Prof. Fangbiao Tao’s team for Chinese adolescents (Xu et al., 2020; Yang et al., 2022). This scale has been used in several studies and has good reliability in evaluating Chinese adolescents’ psychological symptoms (Xu et al., 2019; Lu et al., 2022b). The scale consists of 39 items divided into three dimensions: emotional symptoms, behavioral symptoms, and social adaptation difficulties. The presence of psychological symptoms was determined by the total score. Each item was selected based on the participant’s actual situation in the past 3 months. Emotional symptoms, behavioral symptoms, and social adaptation difficulties consisted of 17, 9, and 13 entries, respectively. Psychological symptoms were the sum of scores from 39 entries. A symptom lasting more than 1 week was recorded as a positive result with a score of 1; a symptom lasting less than 1 week was recorded as a score of 0. A total score of ≥8 indicated the presence of psychological symptoms in the participants. The questionnaire has good reliability and validity, with a Cronbach’s *a* of 0.96 (Wu et al., 2015).

### 2.6 Covariates

The covariates investigated in this study include indicators such as Father’s education, Mother’s education, Sleep quality, Frequency of breakfast, and Body mass index (BMI). (1) Father’s education and Mother’s education were categorized into three types, i.e., Elementary school or below, Middle School or High School, and College and above. (2) Sleep quality was based on Pittsburgh’s Sleep Quality Index (BMI). quality was investigated based on the Pittsburgh Sleep Quality Index (PSQI), which, as a commonly used self-assessed sleep quality questionnaire in the world, has good reliability and validity in evaluating participants’ sleep quality (Chang et al., 2021). Participants completed the PSQI questionnaire based on their past month’s performance and scores were calculated using a prescribed scoring method. The PSQI questionnaire consisted of 18 items and 7 factors, including sleep latency, sleep efficiency, subjective sleep quality, duration of sleep at night, use of hypnotic medication, sleep disorders, and daytime dysfunction. The PSQI had a total score of 21, with higher scores indicating relatively poorer quality of sleep. The higher the participant’s score, the poorer the relative quality of sleep. Based on the participants’ final scores, sleep quality was categorized as Good (≤5 points), Moderate (6–7 points), and Poor (>7 points). (3) The frequency of breakfast was evaluated by the participants themselves. The frequency of breakfast in the past 7 days was investigated. Based on the results of the self-assessment, participants were categorized into ≤1 time/week, 2–5 times/week, and ≥ 6 times/week. (4) Body mass index (BMI) was calculated based on the results of the height and weight tests, and BMI = weight (kg)/height (m)^2^. The methods of height and weight tests were based on the National Survey on Students’ Constitution and Health. National Survey on Students’ Constitution and Health (CNSSCH) (CNSSCH Association, 2022). Height test results are accurate to 0.1 centimeters. Weight test results are accurate to 0.1 kg. According to the BMI value, they are categorized into Slimmer (BMI≦18.4 kg/m^2^), Normal (18.5–23.9 kg/m^2^), Overweight (24.0–27.9 kg/m^2^), and Obese (≧28 kg/m^2^) (Sharashova et al., 2014).

### 2.7 Quality control

**Figure 2:**
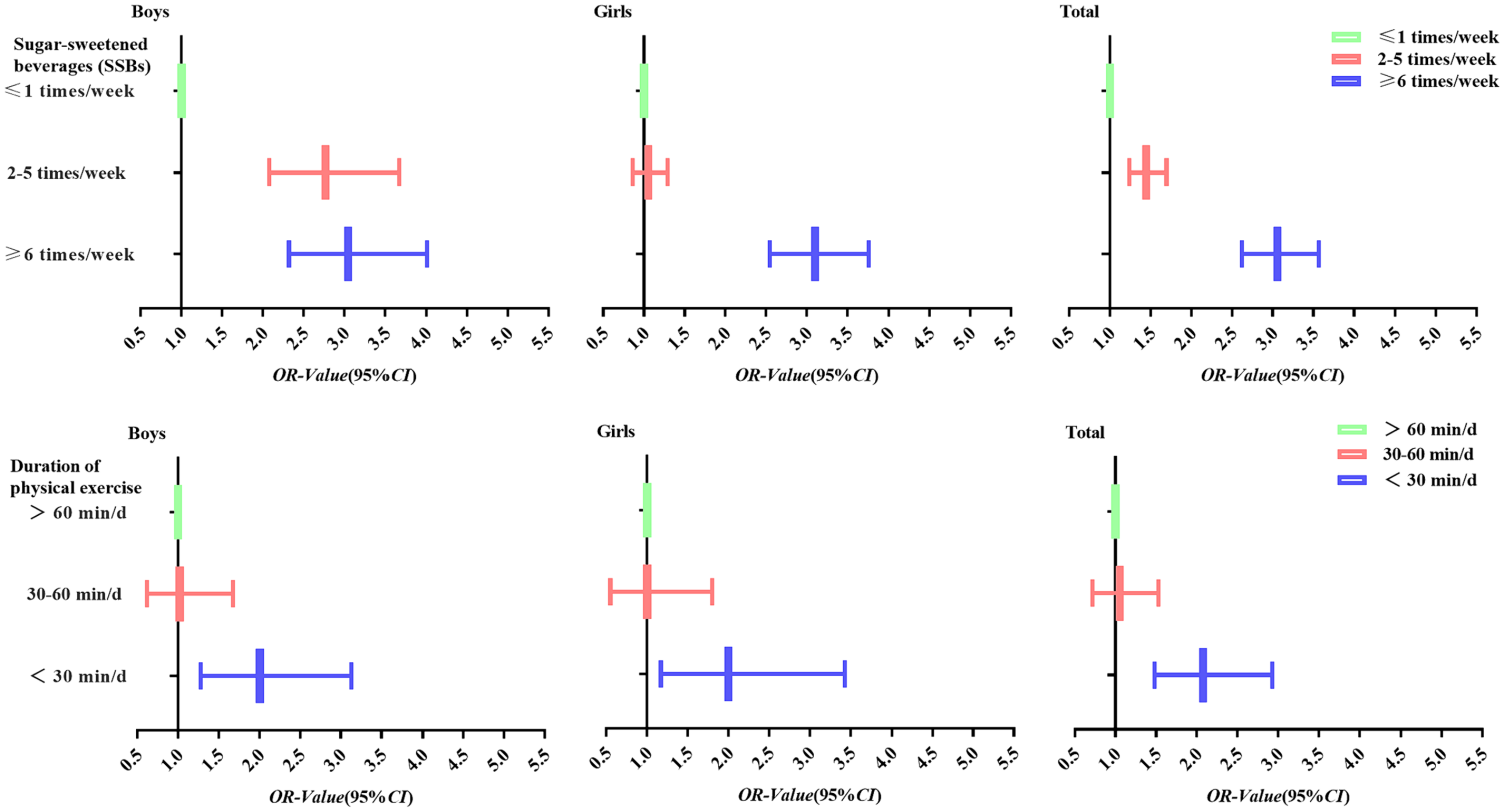
Trends of logistic regression ORs of SSBs, duration of physical exercise, and psychological symptoms among Tibetan university students in high altitude areas of China.

The survey of the self-assessment questionnaire of this study was conducted by trained professional teachers as survey staff, who entered the participants’ classes one by one to conduct the questionnaire. The purpose and requirements of this study’s survey were explained to the participants by the survey staff before the survey. The questionnaires were distributed and asked to be filled out independently by the participants, and the questionnaires were retrieved on the spot after filling out the questionnaires. When the questionnaires were withdrawn, the staff checked the completion of the questionnaires one by one and asked the participants to complete the questionnaires on time if they were wrongly filled in or omitted from the questionnaires. For the height and weight tests, participants were asked to empty their bowels and urine before the test to ensure the accuracy of the weight test. For height, students were asked to take off their shoes. Test values were filled in by a staff member on the participant’s questionnaire. The height and weight tests were completed by a teacher who specialized in testing and required that the testing instruments be calibrated before each day’s testing to guarantee the accuracy of the results (Figure 2).

### 2.8 Statistical analysis

The basic situation of Tibetan university students in high altitude areas and the analysis of each variable used percentages (%) to present specific results. The one-way analysis of the association between SSBs and duration of physical exercise and psychological symptoms and the three dimensions among Tibetan university students at high altitudes was conducted by using the chi-square test. The analysis of the association between high altitude Tibetan university students’ SSBs and duration of physical exercise and psychological symptoms was analyzed using Logistic regression analysis. The Crude Model was the initial association analysis without adjusting for any covariates. Model 1 was analyzed based on the Crude model with adjustments for age, father’s education, and mother’s education. Model 2 was analyzed based on model 1 with further adjustments for sleep quality, frequency of breakfast, and BMI. Logistic regression analyses were performed after stratifying by gender.

Data analysis was performed using SPSS 25.0 (SPSS Inc., Chicago, IL, USA) software. A two-sided test level of *α* = 0.05 was used.

## 3 Results

In this study, a total of 8,268 Tibetan university students aged 19–22 years old in high-altitude areas of China were surveyed with self-assessment questionnaires.

The detection rate of psychological symptoms among Tibetan university students at high altitudes in China was 16.7%. The detection rates of Emotional symptoms, Behavioral symptoms, and Social adaptation difficulties were 18.2, 18.4, and 15.1%, respectively. The detection rates of emotional symptoms, behavioral symptoms, and social adaptation difficulties were 18.2, 18.4, and 15.1%, respectively, and the proportions of SSBs of ≤1 times/week, 2–5 times/week, and ≥ 6 times/week were 54.2, 24.3, and 21.5%, respectively. In particular, the proportion of boys (25.0%) with SSBs ≥6 times/week was higher than that of girls (18.8%), and the difference was statistically significant (*χ*^2^ = 29.36, *p* < 0.001). Duration of physical exercise was >60 min/d was only 5.4%.

For each covariate, the proportion of the Father’s education in college and above (8.4%) was higher than the proportion of the mother’s education for college and above (4.8%), and the difference was statistically significant in comparison (*χ*^2^ = 77.19, *p* < 0.001). The proportion of Tibetan university students at high altitudes whose Sleep quality was Poor (>7 points) was 62.8%, and the proportion of girls students (66.5%) was higher than that of boys students (57.9%). The proportion of high-altitude university students whose frequency of breakfast was ≤1 time/week was 11.9%. The proportions of Tibetan university students with BMI of slimmer, normal, overweight, and obese in high-altitude areas of China were 17.0, 53.1, 12.5, and 17.4%, respectively. The proportion of obese students was higher for male students (18.3%) than for female students (16.6%) (Table 1).

**Table 1:** Basic status of Tibetan university students in high-altitude areas of China (%).

Categorization	Boys	Girls	Total
Number (N)	3,506	4,762	8,268
Age			
19 yrs	1,010 (28.8)	1,722 (36.2)	2,732 (33)
20 yrs	1,070 (30.5)	1,569 (32.9)	2,639 (31.9)
21 yrs	789 (22.5)	978 (20.5)	1,767 (21.4)
22 yrs	637 (18.2)	493 (10.4)	1,130 (13.7)
Father’s education			
Elementary school or below	1,053 (30.0)	1,272 (26.7)	2,325 (28.1)
Middle school or high school	2,185 (62.3)	3,065 (64.4)	5,250 (63.5)
College and above	268 (7.6)	425 (8.9)	693 (8.4)
Mother’s education			
Elementary school or below	1,638 (46.7)	2,153 (45.2)	3,791 (45.9)
Middle school or high school	1,687 (48.1)	2,396 (50.3)	4,083 (49.4)
College and above	181 (5.2)	213 (4.5)	394 (4.8)
Sleep quality			
Good (≤5 points)	1,101 (31.4)	1,060 (22.3)	2,161 (26.1)
Moderate (6–7 points)	376 (10.7)	535 (11.2)	911 (11.0)
Poor (>7 points)	2,029 (57.9)	3,167 (66.5)	5,196 (62.8)
Frequency of breakfast			
≤1 times/week	593 (16.9)	392 (8.2)	985 (11.9)
2–5 times/week	1,039 (29.6)	1,082 (22.7)	2,121 (25.7)
≥6 times/week	1,874 (53.5)	3,288 (69.0)	5,162 (62.4)
Body mass index (BMI)			
Slimmer	385 (11.0)	1,024 (21.5)	1,409 (17.0)
Normal	1,810 (51.6)	2,577 (54.1)	4,387 (53.1)
Overweight	668 (19.1)	369 (7.7)	1,037 (12.5)
Obese	643 (18.3)	792 (16.6)	1,435 (17.4)
SSBs			
≤1 times/week	1,910 (54.5)	2,571 (54)	4,481 (54.2)
2–5 times/week	719 (20.5)	1,294 (27.2)	2,013 (24.3)
≥6 times/week	877 (25.0)	897 (18.8)	1,774 (21.5)
Duration of physical exercise			
<30 min/d	2,347 (66.9)	3,917 (82.3)	6,264 (75.8)
30–60 min/d	862 (24.6)	696 (14.6)	1,558 (18.8)
>60 min/d	297 (8.5)	149 (3.1)	446 (5.4)
Psychological symptoms			
Emotional symptoms	574 (16.4)	928 (19.5)	1,502 (18.2)
Behavioral symptoms	603 (17.2)	917 (19.3)	1,520 (18.4)
Social adaptation difficulties	535 (15.3)	715 (15.0)	1,250 (15.1)
Psychological symptoms	519 (14.8)	865 (18.2)	1,384 (16.7)

The results of this study showed that, overall, the detection rates of psychological symptoms in Tibetan university students at high altitudes in China were compared in terms of different SSBs and duration of physical exercise, and the differences were all statistically significant (*χ*^2^ = 560.0, 117.2, *p* < 0.001). In terms of different dimensions, the differences in the detection rates of emotional symptoms among different SSB consumption frequency and duration of physical exercise university students were also statistically significant when compared (*χ*^2^ = 767.52, 106.60, *p* < 0.001); in Behavioral symptoms, the differences were also statistically significant (*χ*^2^ = 1109.22, 60.17, *p* < 0.001); in social adaptation difficulties, the differences were also statistically significant (*χ*^2^ = 1642.76, 17.97, *p* < 0.001).

For the consumption of different SSBs consumption frequency, the differences in the detection rates of emotional symptoms, behavioral symptoms, social adaptation difficulties, and psychological symptoms among boys were statistically significant (*p* < 0.001). The same trend was found in girls. The results of the univariate analyses of the duration of physical exercise with psychological symptoms and the dimensions, for different genders, are shown in Table 2.

**Table 2:** Univariate analysis on SSBs and duration of physical exercise and psychological symptoms of Tibetan university students in high-altitude areas (%).

Categorization		N	Emotional symptoms			Behavioral symptoms			Social adaptation difficulties			Psychological symptoms		
			N (%)	*χ*^2^-value	*P*-value	N (%)	*χ*^2^-value	*P*-value	N (%)	*χ*^2^-value	*P*-value	N (%)	*χ*^2^-value	*P*-value
Boys														
SSBs	≤1 times/week	1910	119 (6.2)	379.49	<0.001	86 (4.5)	621.83	<0.001	54 (2.8)	576.01	<0.001	113 (5.9)	301.43	<0.001
	2–5 times/week	719	146 (20.3)			142 (19.7)			155 (21.6)			139 (19.3)		
	≥6 times/week	877	309 (35.2)			375 (42.8)			326 (37.2)			267 (30.4)		
Duration of physical exercise	<30 min/d	2,347	456 (19.4)	51.00	<0.001	437 (18.6)	10.25	0.006	396 (16.9)	14.29	0.001	423 (18.0)	58.37	<0.001
	30–60 min/d	862	79 (9.2)			121 (14.0)			103 (11.9)			71 (8.2)		
	>60 min/d	297	39 (13.1)			45 (15.2)			36 (12.1)			25 (8.4)		
Girls														
SSBs	≤1 times/week	2,571	325 (12.6)	441.74	<0.001	283 (11.0)	530.39	<0.001	71 (2.8)	1133.29	<0.001	325 (12.6)	312.86	<0.001
	2–5 times/week	1,294	205 (15.8)			221 (17.1)			201 (15.5)			194 (15.0)		
	≥6 times/week	897	398 (44.4)			413 (46)			443 (49.4)			346 (38.6)		
Duration of physical exercise	<30 min/d	3,917	835 (21.3)	47.78	<0.001	831 (21.2)	54.64	<0.001	610 (15.6)	5.79	0.055	783 (20.0)	49.59	<0.001
	30–60 min/d	696	73 (10.5)			69 (9.9)			89 (12.8)			66 (9.5)		
	>60 min/d	149	20 (13.4)			17 (11.4)			16 (10.7)			16 (10.7)		
Total														
SSBs	≤1 times/week	4,481	444 (9.9)	767.52	<0.001	369 (8.2)	1109.22	<0.001	125 (2.8)	1642.76	<0.001	438 (9.8)	560.00	<0.001
	2–5 times/week	2013	351 (17.4)			363 (18.0)			356 (17.7)			333 (16.5)		
	≥6 times/week	1774	707 (39.9)			788 (44.4)			769 (43.3)			613 (34.6)		
Duration of physical exercise	<30 min/d	6,264	1,291 (20.6)	106.60	<0.001	1,268 (20.2)	60.17	<0.001	1,006 (16.1)	17.97	<0.001	1,206 (19.3)	117.20	<0.001
	30–60 min/d	1,558	152 (9.8)			190 (12.2)			192 (12.3)			137 (8.8)		
	>60 min/d	446	59 (13.2)			62 (13.9)			52 (11.7)			41 (9.2)		

In this study, logistic regression was performed with Tibetan university students’ psychological symptoms as the dependent variable and SSBs consumption frequency and duration of physical exercise as the independent variables in high-altitude areas of China. After adjusting for the relevant covariates, the results of Model2 showed that overall, compared with university students in the SSBs ≤1 times/week group, SSBs 2–5 times/week (OR = 1.45, 95%) were more likely than SSBs ≤1 times/week (OR=1.45, 95% CI: 1.24–1.70) and ≥6 times/week (OR=3.06, 95% CI: 2.62–3.57) university students had an increased risk of psychological symptoms (*p* < 0.001). Regarding the duration of physical exercise, the reference group was the duration of physical exercise >60 min/d, and the university students in the Duration of Physical Exercise <30 min/d (OR = 2.08, 95% CI: 1.48–2.93) group had an increased risk of developing psychological symptoms (*p* < 0.001). University students in the group also had a significantly increased risk of psychological symptoms (*p* < 0.001). The results of logistic regression analysis in terms of different genders are shown in Table 3.

**Table 3:** SSBs and duration of physical exercise and psychological symptoms of Tibetan university students in high-altitude areas Logistic regression analysis (*N* = 8,268).

Sex	Categorization		Crude model		Model 1		Model 2	
			OR (95% CI)	*P -*value	OR (95% CI)	*P -*value	OR (95% CI)	*P -*value
Boys	SSBs	≤1 times/week	1.00		1.00		1.00	
		2–5 times/week	3.81 (2.92 ~ 4.97)	<0.001	3.67 (2.81 ~ 4.79)	<0.001	2.77 (2.08 ~ 3.67)	<0.001
		≥6 times/week	6.96 (5.48 ~ 8.83)	<0.001	6.43 (5.05 ~ 8.18)	<0.001	3.05 (2.32 ~ 4.01)	<0.001
	Duration of physical exercise	>60 min/d	1.00		1.00		1.00	
		30–60 min/d	0.98 (0.61 ~ 1.57)	0.922	1.04 (0.64 ~ 1.68)	0.884	1.02 (0.62 ~ 1.68)	0.946
		<30 min/d	2.39 (1.57 ~ 3.65)	<0.001	2.48 (1.62 ~ 3.8)	<0.001	2.01 (1.28 ~ 3.13)	0.002
Girls	SSBs	≤1 times/week	1.00		1.00		1.00	
		2–5 times/week	1.22 (1.01 ~ 1.48)	0.043	1.17 (0.97 ~ 1.42)	0.110	1.05 (0.86 ~ 1.29)	0.610
		≥6 times/week	4.34 (3.63 ~ 5.18)	<0.001	4.09 (3.41 ~ 4.9)	<0.001	3.1 (2.55 ~ 3.76)	<0.001
	Duration of physical exercise	>60 min/d	1.00		1.00		1.00	
		30–60 min/d	0.87 (0.49 ~ 1.55)	0.639	0.83 (0.47 ~ 1.49)	0.539	1 (0.55 ~ 1.8)	0.990
		<30 min/d	2.08 (1.23 ~ 3.51)	0.006	1.87 (1.1 ~ 3.17)	0.021	2 (1.17 ~ 3.43)	0.012
Total	SSBs	≤1 times/week	1.00		1.00		1.00	
		2–5 times/week	1.83 (1.57 ~ 2.13)	<0.001	1.72 (1.48 ~ 2.01)	<0.001	1.45 (1.24 ~ 1.70)	<0.001
		≥6 times/week	4.87 (4.24 ~ 5.6)	<0.001	4.73 (4.1 ~ 5.45)	<0.001	3.06 (2.62 ~ 3.57)	<0.001
	Duration of physical exercise	>60 min/d	1.00		1.00		1.00	
		30–60 min/d	0.95 (0.66 ~ 1.37)	0.794	0.97 (0.67 ~ 1.4)	0.868	1.05 (0.72 ~ 1.53)	0.798
		<30 min/d	2.36 (1.7 ~ 3.27)	<0.001	2.23 (1.6 ~ 3.11)	<0.001	2.08 (1.48 ~ 2.93)	<0.001

The Crude Model was the initial association analysis without adjusting for any covariates. Model 1 was analyzed based on the Crude model with adjustments for age, father’s education, and mother’s education. Model 2 was analyzed based on model 1 with further adjustments for sleep quality, frequency of breakfast, and BMI.

## 4 Discussion

Various psychological problems of university students are constantly emerging and adversely affecting their studies at the university level and their future employment after graduation (Gardani et al., 2022). However, more studies have focused on the psychological symptoms of university students in the plains, while fewer studies have been conducted on Tibetan university students living in harsh natural environments at high altitudes (Acharya et al., 2018). Previous studies have confirmed that psychological symptoms among university students of different ethnicities vary greatly depending on their living environment and lifestyle (Chen et al., 2022). China’s Qinghai-Tibetan Plateau region is a typical high-altitude region in the world, and Tibetans have been living in this region for generations. The investigation and cause analysis of psychological symptoms among Tibetan university students are of great significance in promoting the development of this group’s psychological health. The results of this study showed that the detection rate of psychological symptoms among Tibetan university students in high-altitude areas of China was 16.7%, and it was higher among female students (18.2%) than male students (14.8%). This result compares favorably with Wang et al.’s findings on Chinese university students in the plains (8.1%) (Wang et al., 2022). The reason may be the fact that Tibetans have been living at high altitudes for generations and are better adapted to the natural environment at high altitudes may also be an important reason for the lower psychological symptoms.

The results of this study also showed that the proportion of Tibetan university students with SSBs ≥6 times/week in high-altitude areas of China was 21.5%. This suggests that SSBs among Tibetan university students in high-altitude areas are worthy of concern and attention. Some studies have confirmed that excessive SSBs will lead to the occurrence of various chronic cardiovascular diseases, such as hypertension, diabetes, and other diseases (Qin et al., 2020). At the same time, an excess of SSBs can lead to obesity, which is an important risk factor for psychological problems (Ra, 2022). Therefore, effective control of SSBs among university students is important for the prevention of various diseases and psychological disorders. In addition, the results of this study confirmed that only 5.4% of Tibetan university students in high-altitude areas of China had a duration of physical exercise greater than 60 min/d. This result is consistent with the findings of many previous studies that the lack of physical activity among university students is significant (Smout et al., 2023). The results of this study also indicate that the problem of insufficient physical exercise among university students does not change depending on the altitude. A study confirms that there is a strong association between the duration of physical exercise and academic performance and psychological well-being among university students and that active physical activity has a positive effect on academic performance and psychological well-being (Nie et al., 2023).

The results of this study also confirmed that excess SSBs were positively associated with the development of psychological symptoms, i.e., the higher the number of SSBs, the higher the risk of developing psychological symptoms. In a study of Australian adolescents, SSBs were positively associated with the development of depression and anxiety (Smout et al., 2023). Another study of Chinese adolescents also confirmed that SSBs were significantly associated with the development of psychological symptoms in adolescents (Zhang et al., 2022). Research has confirmed that there is a strong correlation between corticotropin-releasing hormone (CRH), galanin (GAL), and mood swings and that there is a strong correlation between hormonal changes in the body and SSBs (Rana et al., 2022). The results of this study also confirmed that there is a close association between the duration of physical exercise and psychological symptoms among Tibetan university students in high-altitude areas of China, and the shorter the duration of physical exercise, the higher the risk of psychological symptoms among the shorter the duration of physical exercise, the higher the risk of psychological symptoms among university students. Several studies have confirmed that physical exercise in adolescents has an important positive effect on the promotion of mental health, and also plays a positive role in the prevention of psychological symptoms (Grasdalsmoen et al., 2020; Herbert, 2022). In terms of gender, there was a significant association between SSBs and duration of physical exercise and psychological symptoms. It shows that this relationship does not change according to gender.

The results of this study may provide some guidance for future mental health interventions and promotion for Tibetan university students in high-altitude areas of China. This study has some strengths and limitations. In terms of strengths, first, this study analyzed for the first time the association between SSBs and duration of physical exercise and psychological symptoms among Tibetan university students at high altitudes in China, which may provide guidance for future mental health interventions and promotion for Tibetan university students at high altitudes. The present study analyzed the association between SSBs and duration of physical exercise and psychological symptoms for the first time in China, which can help to promote the psychological health of Tibetan university students at high altitudes in the future. Second, this study adjusted the covariates when analyzing the relationship between SSBs and duration of physical exercise and psychological symptoms to make the results more objective and realistic. Third, the sample size of this study is relatively large in high-altitude studies, and the results are representative. However, this study also has some limitations. On the one hand, the surveys of SSBs, duration of physical exercise, and psychological symptoms in this study were conducted using self-assessment questionnaires, and there may be some deviations between the results and the real situation. In the future, more accurate assessment methods should be used to conduct related surveys. On the other hand, this study is a cross-sectional survey, which can only analyze the association between SSBs and duration of physical exercise and psychological symptoms, but cannot analyze the causal relationship between them. Future cohort studies in this population are needed to further analyze the causal relationship.

## 5 Conclusion

The results of this study show that there is an association between SSBs and duration of physical exercise and psychological symptoms among Tibetan university students at high altitudes in China. SSBs in excess and insufficient duration of physical exercise may be important risk factors for the development of psychological symptoms among Tibetan university students at high altitudes. Sweetened beverages and duration of physical exercise may be important risk factors for the development of psychological symptoms in Tibetan university students at high altitudes. The findings of this study may provide a possible reference for future mental health promotion and intervention for Tibetan university students at high altitudes. It also provides possible guidance and assistance to the education and administrative departments of high-altitude areas in formulating relevant policies.

## Data availability statement

The raw data supporting the conclusions of this article will be made available by the authors, without undue reservation.

## Ethics statement

The studies involving humans were approved by the Ethics Committee of Jiangxi Science and Technology Normal University (IRB-JXSTNU-2022003). The studies were conducted in accordance with the local legislation and institutional requirements. The participants provided their written informed consent to participate in this study.

## Author contributions

WS: Conceptualization, Investigation, Resources, Supervision, Validation, Visualization, Writing – original draft, Writing – review & editing. FS: Conceptualization, Investigation, Visualization, Writing – original draft. SL: Data curation, Funding acquisition, Investigation, Writing – original draft. YS: Formal analysis, Project administration, Software, Writing – original draft, Writing – review & editing. GC: Funding acquisition, Writing – original draft.
